# Bio and Geno-toxic Activities of Cadmium- Arsenic Salts Combination And/or Fluoride in Female Rats Confirmed by Molecular Docking

**DOI:** 10.1007/s12011-025-04972-9

**Published:** 2026-02-24

**Authors:** Doaa S. Foda, Noha E. Ibrahim

**Affiliations:** 1 Therapeutic Chemistry Department, Pharmaceutical and Drug Industries Research Institute, National Research Centre (NRC), 33 El-Buhouth St, Dokki, Giza, P.O. 12622 Egypt; 2 Microbial Biotechnology Department, Biotechnology Research Institute, National Research Centre (NRC),33 El-Buhouth St, Dokki, Giza, P.O.12622 Egypt

**Keywords:** Sodium fluoride, Heavy metals combination, Biochemical and genotoxic effects, Docking

## Abstract

Heavy metals are increasingly recognized as major toxic agents and potential carcinogens. This research was designed to assess the biochemical and genetic toxicity that arises from the exposure to a combination of cadmium and arsenic salts and/or fluoride in rats over a period of two months. Additionally, this study examined the molecular interactions of these salts with two crucial target proteins: heat shock protein 70 (HSP70) and metallothionein (MT1) through molecular docking analysis. Rats were divided into four groups. Group 1: rats were administrated sodium fluoride only. Group 2: rats received a combination between cadmium and arsenic salts. Group 3: rats were administrated mixture of the combination between cadmium and arsenic salts in addition to sodium fluoride. Group 4: rats represented the normal control. Biochemical and molecular as well as docking studies in addition to histological examinations were evaluated. Each rat group exhibited unique biochemical findings (in comparison to the control group) that were different from those of the other groups. Noticeable significant alterations in serum thyroid hormones (FT4 and FT3) were found in the group that received only fluoride (F group) associated with a predicted later cardiac dysfunction. Unusual significant reduction in triglyceride levels was observed in the rats treated with the combination of heavy metals (H group) which may contribute to a case of cardiomyopathy. On the other hand, the group receiving the mixture of all three salts (H + F) group showed biochemical results reaching normal levels. Thrombocytosis, characterized by increased platelet counts, was observed in the (F), (H), and (H + F) groups. At the molecular level, the results obtained from the PCR technique indicated that the highest level of damage was observed in the group that received the mixture of heavy metals and fluoride (H + F). This group showed a significantly reduced gene expression of (HSP70) and (MT1) in liver tissues. Docking investigations showed that sodium fluoride, cadmium chloride, and sodium arsenate have unique but complementary binding mechanisms with (HSP70) and (MT1). Sodium fluoride stabilizes connections by hydrogen bonds and hydrophobic contacts, whereas cadmium chloride uses metal coordination and pi-alkyl networks. Sodium arsenate, with its high-affinity hydrogen bonding, further enhances binding stability. Despite achieving normal levels in its specified biochemical parameters, the group exposed to the mixture of heavy metals and fluoride (H + F) experienced more harmful effects and worsened overall health compared to the groups administrated fluoride or heavy metals combination alone. This was attributed to the suppression of essential cellular defense mechanisms and a rise in platelet (PLT) count which in turn lead to an accelerated and increased mortality.

**Supplementary Information:**

The online version contains supplementary material available at 10.1007/s12011-025-04972-9.

## Introduction

New generations now face a growing array of environmental hazards that silently impact their health. Over the past decades, studies have reported the accumulation of toxic metals and non-metals in water, plants, farm animals, marine organisms, and the resulting food chain [1, 2].

Two of the most toxic elements that threaten human’s life are cadmium (Cd) and arsenic (As) despite of their relatively low natural abundance [3–5]. This is attributed to the industrial revolution, which has led to an intractable problem of heavy metal waste and accumulation in the surrounding environment [6–8].

Cadmium ions reach the environment through wastewater from pigment, plastic, electroplating, and zinc refining production. Volcanic activity and human practices like burning also contribute cadmium to soils [9–11]. Arsenic pollution stems from oil combustion, mining, and the use of arsenic-containing pesticides, rodenticides, and herbicides [12–14].

Cadmium is a non-biodegradable cation that can persist in soils for decades due to its long biological half-life, which can exceed 20 years [15, 16]. It has no essential biological use and exhibits low excretion rates when ingested by animals or humans. It primarily targets the liver at high doses and the kidneys at low doses [17]. Long-term exposure can lead to deleterious effects on body organs, initiating various tissue cancers due to its genotoxic impact on DNA and RNA formation, as well as enhancing oxidative stress [18, 19]. Consequently, the International Agency for Research on Cancer (IARC) has classified cadmium as a Category I carcinogen for humans [20].

One should be careful when consuming fish and marine organisms as they are known to be good bioaccumulators of cadmium [21].

The researchers’ attention was also drawn to study the toxicity of arsenic (As) due to its complex chemistry. Arsenic is considered a metalloid, as it exhibits both metallic and non-metallic properties depending on its chemical form and oxidation state [22]. Arsenic can be found in inorganic (highly toxic) or organic (low toxicity) salt forms [23, 24].

For many years, (As) was used as an additive in poultry feed in some countries to prevent diseases. However, this practice has been recently discontinued [25–27]. The dangerous effect of these arsenic additives was an increase in (As) concentration in poultry meat, leading to higher (As) levels in populations consuming this meat [28]. (As) also was used as a therapy for some human diseases before the widespread use of antibiotic drugs [29].

In vivo research has shown that (As), when acting as a pollutant, primarily attacks antioxidants, selenium-dependent enzymes, and thiol groups in biological systems, resulting in increased oxidative stress [30, 31]. High-level exposure to (As) can lead to epigenetic changes, such as increased DNA methylation through the tumor protein P53 and interference with DNA repair systems, classifying it as a type I carcinogen [32, 33]. Arsenic exposure has been linked to various diseases, including neurological, cardiovascular, and skin lesions that can progress to skin cancer. The toxic effects of (As) are further exacerbated by alcohol consumption and cigarette smoking.

Another potentially hazardous element for human health is fluorine (F), a non-metallic element that naturally occurs in soil, groundwater, and some plants. The dissolution of minerals plays a significant role in its presence in the environment [34]. Fluorine is often added to drinking water in some countries [35–38] for maintaining healthy teeth and bones and is used in dental medicine to prevent tooth decay. However, high doses of fluoridated water can lead to its accumulation in the soil, plants, and farm animals, resulting in numerous complications, such as bone fractures, thyroid disorders, joint paralysis, and dysfunction of the brain, cardiovascular system, liver, kidneys, and adrenal glands [39–41]. Some plants and crops, like tea leaves and certain edible crops, can naturally accumulate fluoride from the soil [42, 43].

It is evident from the earlier research that cadmium, arsenic, and fluoride, when given alone, lead to extremely harmful effects on both animals and humans. Nevertheless, our surroundings are burdened with a mixture of various toxic substances and pollutants, to which individuals are continuously exposed.

Consequently, the objective of this research was to examine the risks and complications affecting body organs stemming from the administration of moderate doses of cadmium-arsenic salt combinations and/or fluoride to female rats during a two-month exposure period.

Additionally, the study discussed for the first time the molecular interactions of two significant toxicity-related genes: Heat shock protein 70 (HSP70) and metallothionein (MT1) with each salt administered separately and in combination. This was achieved by using the docking analysis techniques.

## Materials and Methods

### Chemicals and Kits

Sodium fluoride (Na F), Cadmium chloride (CdCl_2_.2H_2_O) and Di-sodium hydrogen arsenate heptahydrate (Na_2_HAsO_4_.7H_2_O) were purchased as a pure quality chemicals from Sigma and Merck, Switzerland.

Kits used for serum biochemical determinations and RT-PCR technique were of a high chemical grade and obtained from Biodiagnostic company, Egypt and Thermo-scientific company, USA respectively.

### Animals

Forty female Wistar albino rats weighing about 100 g were obtained from the animal house unit of the National Research Centre, Giza, Egypt. The animals were housed under standard laboratory conditions (12 h light and 12 h dark) in a room of controlled temperature (24 °C) during the experimental period. The rats were provided *ad libitum* with tap water and fed with standard commercial rat chow.

### Ethical Approval

All the studies were conducted in accordance with the Animal Ethical Committee of the National Research Centre, Dokki, Giza, Egypt under the ethics number (13060139-1).

### Experimental Design

####  Preparation of the Doses

Doses were prepared for oral gavages in accordance to the weight of the rats by dissolving the salts in water to acheive a specific concentration/kgbdwt for each rat in each administration time.

Sodium fluoride solution was prepared by dissolving the salt in water to obtain a concentration of 20 mg/kg bd wt [44].

Cadmium chloride and sodium arsenate combination solution was prepared by adding and dissolving both salts together in the same constant volume of water to obtain the concentrations of 15 mg/kg bd wt for each salt [45, 46].

Cadmium chloride, sodium arsenate and sodium fluoride mixture solution was prepared by adding and dissolving the three powder salts in the same constant volume of water to obtain the concentrations of 15 mg/kg bd wt, 15 mg/kg bd wt and 20 mg/kg bd wt for the previous stated salts respectively.

#### Animal Groups

Rats were divided into 4 groups and administrated orally by gavages a day after the other the following supplementations for two months besides their daily normal diet and water:

- The 1 st group (F group) administrated only sodium fluoride (Na F) solution (20 mg/kg bd wt).
- The 2nd group (H group) administrated water suspension of the combination of cadmium chloride and di-sodium hydrogen arsenate at the doses 15 mg/kg bd wt for each salt.
- The 3rd group (H + F) group administrated water suspension mixture of Na F (20mgkg bd wt) in addition to the combination of CdCl_2_.2H_2_O and Na_2_HAsO_4_.7H_2_O (15 mg/kg bd wt for each salt).
- The 4th group represented the normal control and did not receive any supplementations.

Body weights of each group were determined every month till the end of the experiment.

After two months, rats were prepared for dissection which was preceded by collection of the blood from the retro*-*orbital plexus of the eyes.

1 ml of the blood was kept in EDTA tubes for evaluating the hematological parameters. The remaining blood volume was allowed to settle in sterilized tubes and then centrifuged for 10 min at 4000 r.p.m to obtain the serum that was stored at −20 °C till performing the biochemical assays. Rats were then dissected after their dislocation.Suitable parts from the liver tissues from each group were excised, rinsed with phosphate-buffer saline (PBS) and was immediatly stored at -80 degree celsius for molecular analysis. 

Livers, left kidneys, thyroid glands, and right femur bone (for the extraction of the bone marrow) were removed from each group, cleaned and were rinsed in 10% formalin to perform histological assays.

### Biochemical Studies

Serum enzymatic calorimetric determination of alkaline phosphatase (ALP), alanine and aspartate aminotransferases (ALT and AST), lactate dehydrogenase (LDH) in addition to cholesterol and triglycerides assays were performed [47]. Creatine phosphokinase (CPK) was estimated calorimetrically according to the manufacture kit. Calorimetric determinations of urea and creatinine were also estimated [48].

Serum free thyroxine (fT4) and free tri-iodothyronine (fT3) were determined by ELISA according to the kit instructions.

### Quantitative Real time Polymerase Chain Reaction (qRT-PCR) Test

According to the manufacturer’s instructions, liver tissues were mechanically homogenized (POLYTRON PT 10-35GT, Kinematica AG, Switzerland) in TRIZOL (Thermo Fisher Scientific, USA) to extract total RNA. The NanoDrop 1000 (Thermo Scientific, Wilmington, DE, USA) was used to determine the quantity and quality of RNA. Subsequently, RNAwas treated by RQ1 RNase-Free DNase (Promega, Cat. #M6101), followed by cDNA synthesis using Revert Aid First Strand cDNA Synthesis Kit (Thermo Fisher Scientific, Cat. # K1622) according to the manufacturer’s instructions.

Two genes metallothionine1 and heat shock protein 70 (MT1, and HSP70) were conducted using qPCR (qTOWER3 G Real-Time PCR Thermal Cycler, Germany), and their gene expression was normalized to rat β-Actin.

qPCR was performed using 15 µL of reaction volume, containing 3µL of a 5*X* HOT FIREPoL Solis GreenqPCR Mix(Solis BioDyne, Tartu, Estonia), DNase/RNase-Free water, and 0.3 µM of each primer (Macrogen Co. Ltd., Seoul, Republic of Korea) and 0.75µL of cDNA template.

**Table 1:** Effect of two months administration of heavy metals combination and/or fluoride on hematological parameters in female rats

Groups Parameters	Fluoride (F)	Heavy metals (H)	Heavy metals and fluoride (H+F)	Control
RBCs(10^12^/L)	7.78±0.46*	8.06±0.30*	**7.31±0.47**	**6.84±0.36**
HGB (g/dl)	12.49±0.43*	12.84±0.51*	**11.90±0.65**	**11.63±0.65**
HCT (%)	**42.37±1.67**	47.94±2.52*	38.54±2.17*	**43.90±1.90**
MCV (fl)	54.52±2.93*	59.42±1.22*	52.76±1.84*	**64.12±1.33**
MCH (pg)	16.12±0.83*	15.94±0.30*	16.30±0.44*	**17±0.18**
MCHC (g/l)	295.5±16.7 *	**268±72.9**	308±44.38*	**260±43.20**
PLT (10^9^/l)	**416.75±42.09**	458.6±52.18*	473±65.96*	**392.6±17.90**
MPV (fl)	**5.2±0.25**	4.82±0.13*	**5.22±0.1**	**5.23±0.17**
WBCs (10^9^/l)	**1.13±0.37**	1.84±0.26*	**1.18±0.21**	**0.95±0.27**

Data represented as mean ± S.D. *P* significant at *P *≤ 0.05, P* significant compared to the normal control group

The cycling condition includes an initial activation step at 95 °C for 10 min. Amplification was carried out for 40 cycles with denaturation at 95 °C for 10 s, followed by annealingat 60 °C for 30 s, and extension at 72 °C for 30 s. The sequences of the primers of the selected genes were denoted in the current study [49–51] (Supplementary table (S1)).

### Molecular Docking Simulation

Protein structures for (HSP70) and (MT1) were retrieved from the Protein Data Bank (Supplementary table (S2)) and processed to remove water molecules, ions, and bound ligands; hydrogen atoms were then added to the receptors. Ligands were created using Autodock Vina, with all structures saved in PDB format for docking simulations. Autodock Tools was used to allocate polar hydrogen and Gasteiger charges to receptors.

The grid box dimensions (60 Å × 60 Å × 60 Å) were centered on the active site coordinates derived from structural analysis of the co-crystallized ligands (Supplementary table (S2)). For HSP70 (PDB ID: 7F50), the center was positioned at (−6.27, 13.35, −18.55 Å) based on the nucleotide-binding domain’s ATP/ADP binding pocket [52]. For MT1 (AF-P80297-F1), the grid was centered at (−5.50, 3.64, 7.50 Å), targeting the metal-binding cysteine-rich cluster, informed by known metallothionein coordination sites. Additionally, we conducted a redocking control using the co-crystallized ligand (ANP for HSP70) from PDB ID 7F50. The ligand was extracted, energy-minimized, and redocked using the same parameters (exhaustiveness = 8, num_modes = 9). The root-mean-square deviation (RMSD) between the docked pose and the crystal structure was 1.33 Å, indicating reliable pose prediction. For MT1, due to the absence of a co-crystallized ligand, we used a reference Zn²⁺-bound pose from homologous structures (PDB ID 3MA2) for validation, yielding an RMSD of 1.58 Å. These controls confirm the robustness of our AutoDock Vina.

Docking simulations were performed with Autodock Vina, and the resultant complexes were displayed in Discovery Studio 4.5 to examine 2D hydrogen-bond interactions between receptors and ligands.

### Histopathological Studies

Specimens of the liver, kidney, thyroid and femur bone (for extraction of bone marrow) were fixed immediately in 10% formalin for histological studies. Then the tissues were treated with conventional grades of alcohol and xylol, embedded in paraffin and sectioned at 5 μm thickness. The sections were stained with Hematoxylin and Eosin (H and E) stain for studying the histopathological changes [53].

### Statistical Analysis

SPSS (Statistical Package for the Social Sciences) program version 16 and version 18 were used to analyze statistically the biochemical data and PCR data respectively. Data were represented as mean ± SD. *P* ≤ 0.05 was considered significant as calculated by applying the ANOVA test.

## Results

### Body Weight Determination and Biochemical Results

#### Impact of Different Administrations on Body Weight Gain in Rat Groups

**Fig. 1:**
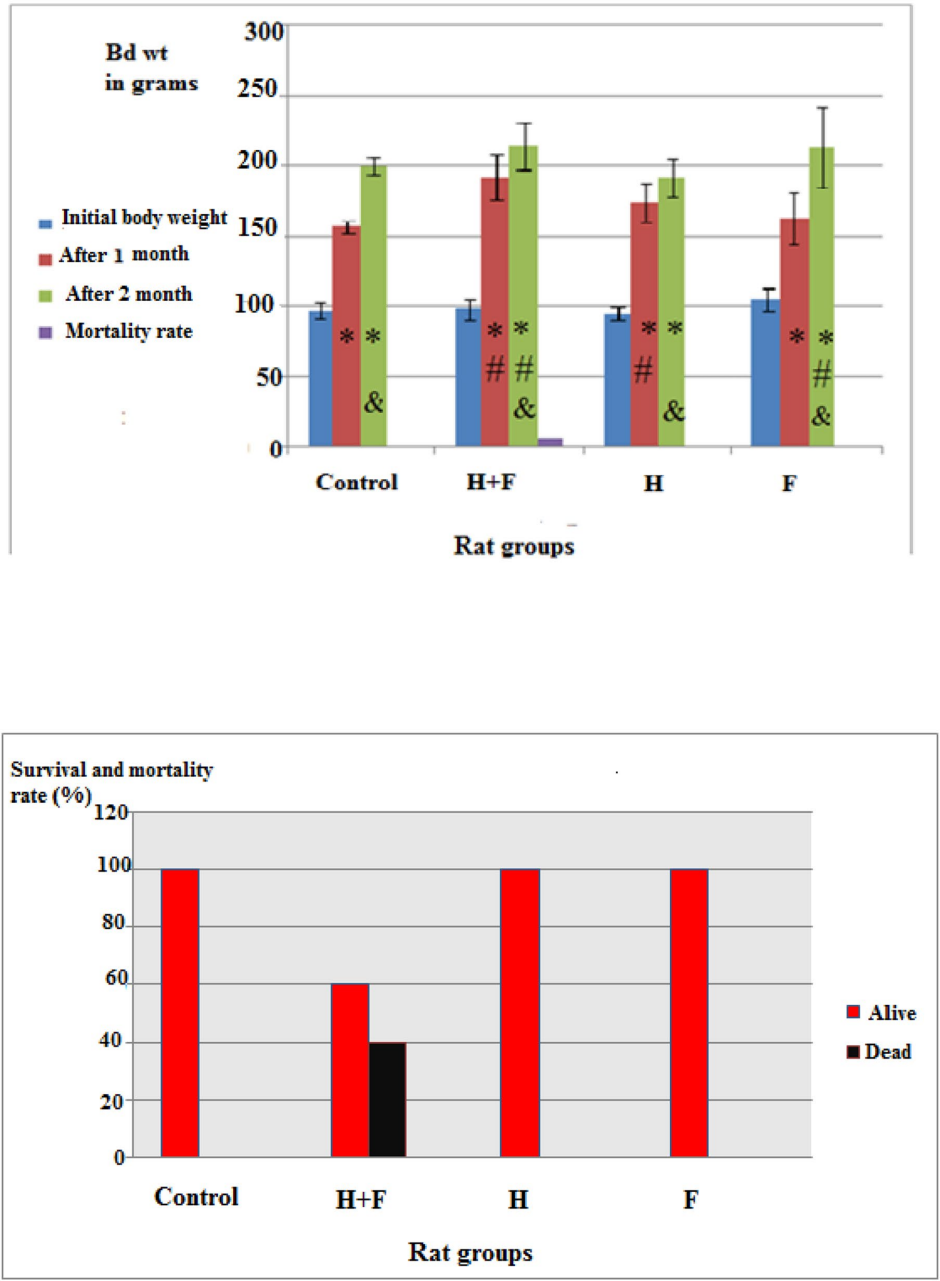
(a) Effect of heavy metal combination and /or fluoride on body weights and mortality rate in female rats. Note: Data represented as mean ± S.D. P significant at P ≤ 0.05, P* significant compared to the corresponding initial weight, P# significant compared to the corresponding final normal control groups, P& significant compared to the corresponding final weight after 1 month. (b) Effect of heavy metal combination and /or fluoride on mortality rate in rat groups. Note: Showing the survival and mortality rates (%) across rat groups during two months of different administrations

**Fig. 2:**
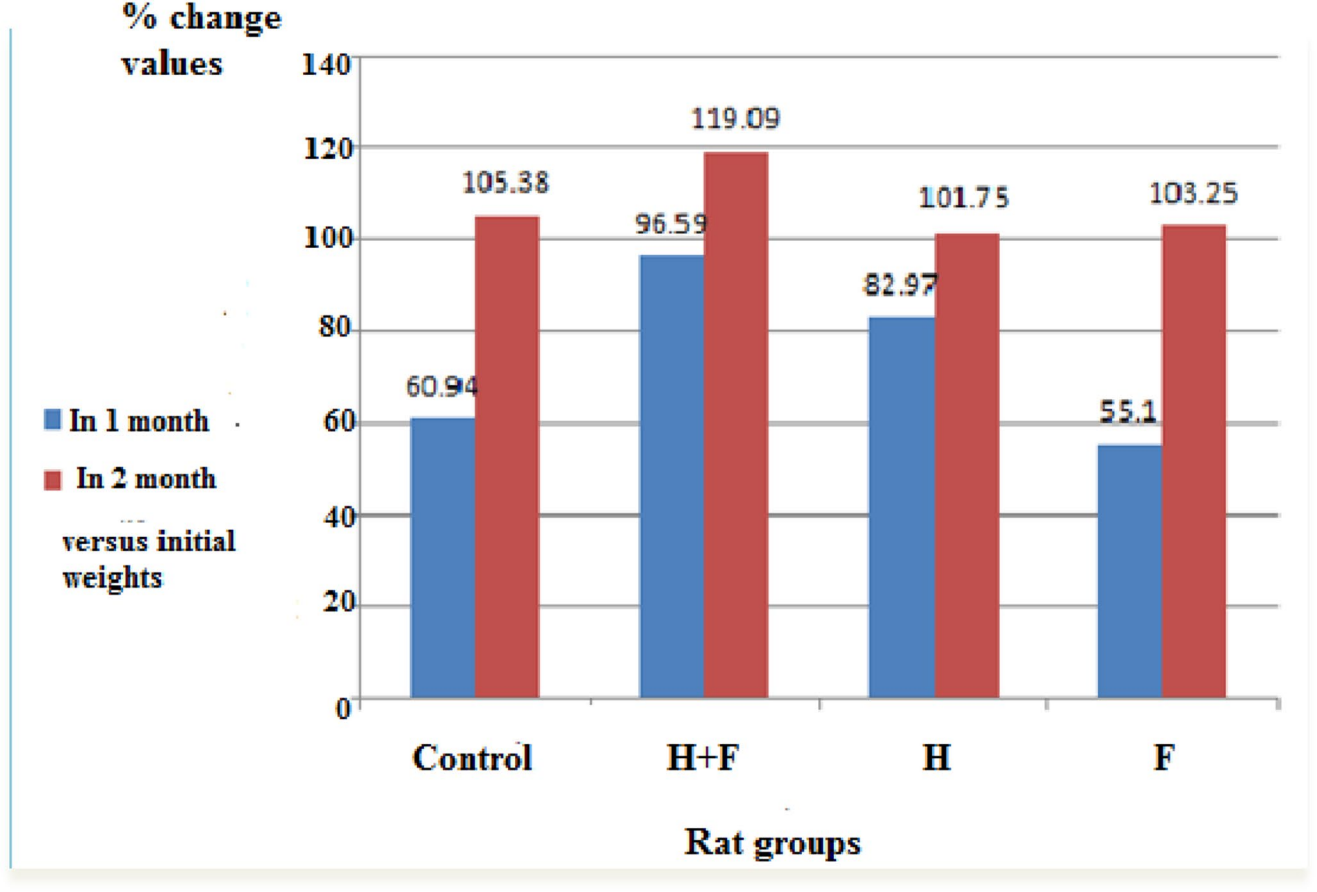
Percent change values in rats' weights after 1 and 2 months representing the percent change in body weights of rats in the different groups compared to their initial corresponding body weights

By examining the body weight change within the groups subjected solely to fluoride and/or the combination of heavy metals (Fig. 1(a) & (2)), it was found that after one month, a statistically significant elevation in body mass across all groups was evident when compared to both their initial corresponding weights and the normal control group.

Subsequent to a two-month administration period, the notable significant enhancement in weight gain persisted within the fluoride-only (F) and the heavy metals combination with fluoride (H + F) administrated groups, compared to the previously mentioned groups.

A marginally retarded, yet statistically non-significant, increase was observed in the heavy metals combination administrated group (H) when compared to the control group, as shown in Fig. 1(a).

The body weight increase following a two-month administration across all groups exhibited a statistically significant enhancement relative to the weight gain recorded after a one-month duration.

Figure 1(a & b) displayed also the mortality rate in rat groups during the two months administration. A mortality rate reached a value equals 40% was observed in the group administrated the mixture of heavy metal combination with fluoride (H+F) (Supplementary table (S3)).

Figure (2) showed the percent change in rats' body weights across the groups after one and two months compared to their corresponding initial weights.

#### Effect of the Different Administrations on Blood Picture

Hematological parameters were affected significantly in the three administrated groups as shown in Table (1). The blood picture parameters appeared to be more affected in (F) and (H) groups more than those determined in (H + F) group compared to the control group.

Platelet count was significantly increased in the three groups. (H + F) group achieved the highest platelet count as well as the highest MCHC value among the groups compared to control group (Table 1).

####  Effect of Heavy Metal and/or Fluoride on the Liver and Kidney Functions in Rat Groups

**Fig. 3:**
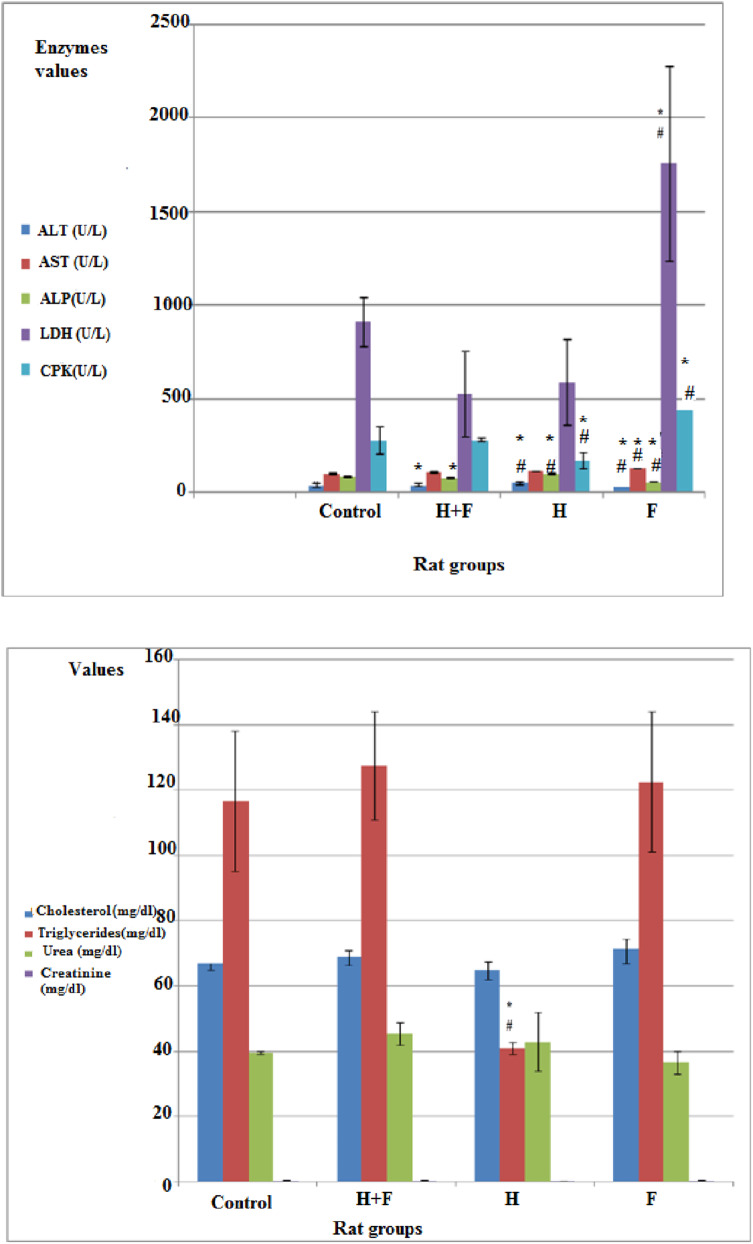
Effect of two months administration of heavy metals combination and/or fluoride on some serum biochemical parameters in female rats. (**a**): Effect of different administrations on some serum enzymes. Footnote: Data was represented as mean ±S.D. *P* significant at *P *≤ 0.05. P* significant compared to the normal control group. *P*^#^ significant compared to (H+F) group. (**b**): Impact of the different administrations on lipid profile and kidney functions. Data is represented as mean ±S.D. *P* significant at *P *≤ 0.05. P* significant compared to the normal control group. *P*^#^ significant compared to (H+F) group

Figures 3(a & b) displayed the serum biochemical parameters to evaluate the liver functions across the various rat groups, serum levels of ALT, AST, ALP, cholesterol, and triglycerides were determined. Furthermore, renal function was assessed via the quantification of serum creatinine and urea. Additionally, serum LDH and CPK levels were documented to predict cardiac functions.

The findings indicated that the most adversely impacted groups were the (F) group and (H) group. A statistically significant elevation was noted in serum concentrations of AST, LDH, and CPK within the (F) group when compared to the control group. This observation suggests a probable onset of cardiac dysfunction, as illustrated in Fig. 3(a). Furthermore, a significant reduction in serum levels of the ALP enzyme was also recorded in the (F) group. As depicted in Fig. 3(b), cholesterol, creatinine, and urea levels remained unchanged across the three groups. Notably, the (H) group achieved the lowest significant level in serum triglycerides among the three administrated groups compared to the normal control group as illustrated in Fig. 3(b) (Supplementary table (S4)).

#### Effect of Heavy Metals and/fluoride on Serum Free Thyroid Hormones in the Different Groups

**Fig. 4:**
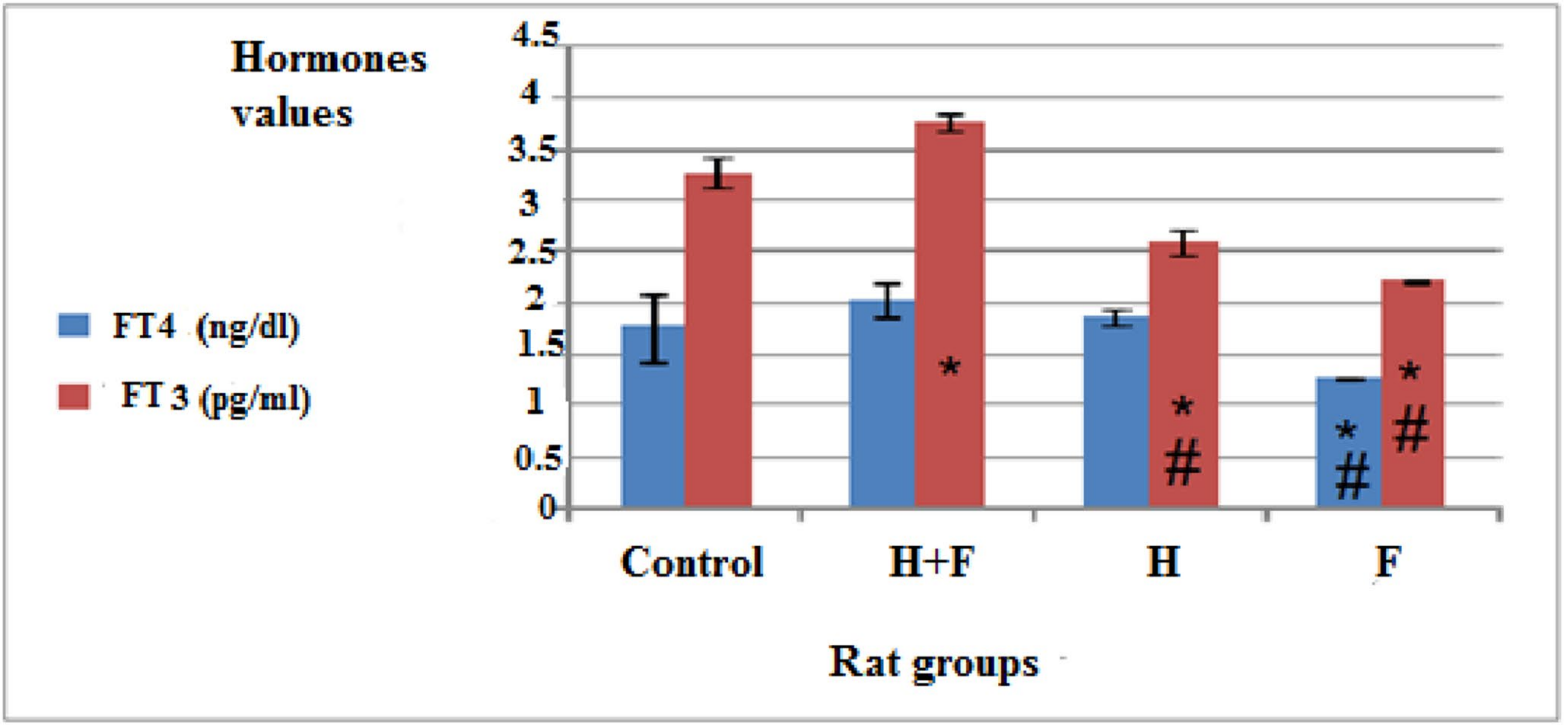
Effect of two months administration of heavy metals combination and/or fluoride on serum FT4 and FT3 in female rats. Data is represented as mean ±S.D. *P* significant at *P *≤ 0.05. P* significant compared to the normal control group. *P*^#^ significant compared to (H+F) group

Thyroid hormones were disrupted in the three administrated groups as shown in Fig. (4). Significant changes were observed in both FT4 and FT3 in the (F) group. This data are considered as an alarm that pointed to a hypothyroidism case initiation. On the other hand, (H) and (H + F) groups achieved significant changes in FT3 only compared to control group (Supplementary table (S5)).

### Molecular results

#### mRNA expression of (MT1) and (HSP70) Genes in Liver Tissue of Different Group

**Fig. 5:**
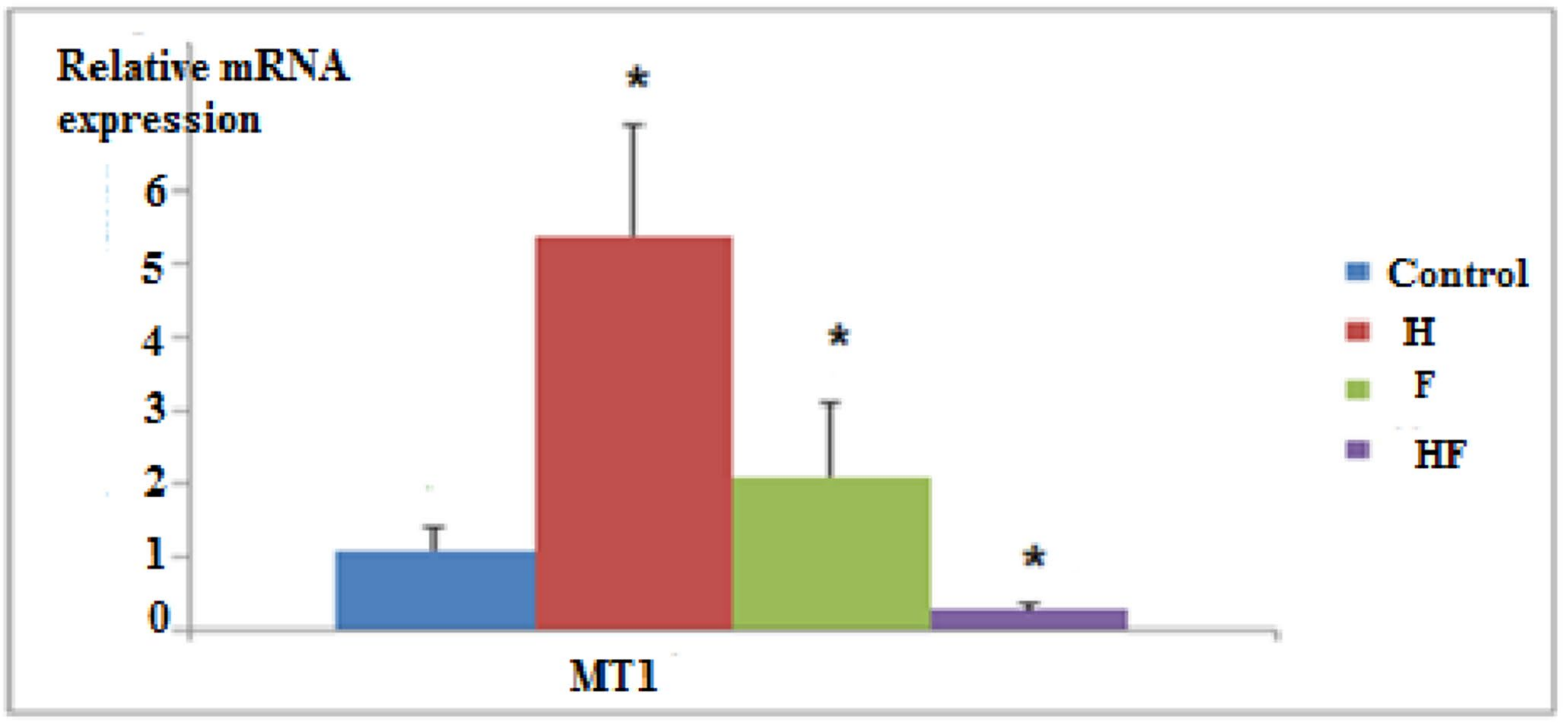
Effect of different administrations on metallothionine 1 (MT1) gene expression level. Data are represented as mean ±SD. *P* significant at *P *≤ 0.05. P* significant compared to the normal control group

Expression of the (MT1) gene was significantly up-regulated in the (H) and (F) groups, while was significantly down-regulated in the HF(H+F) group compared to the control group (Fig. 5).

**Fig. 6:**
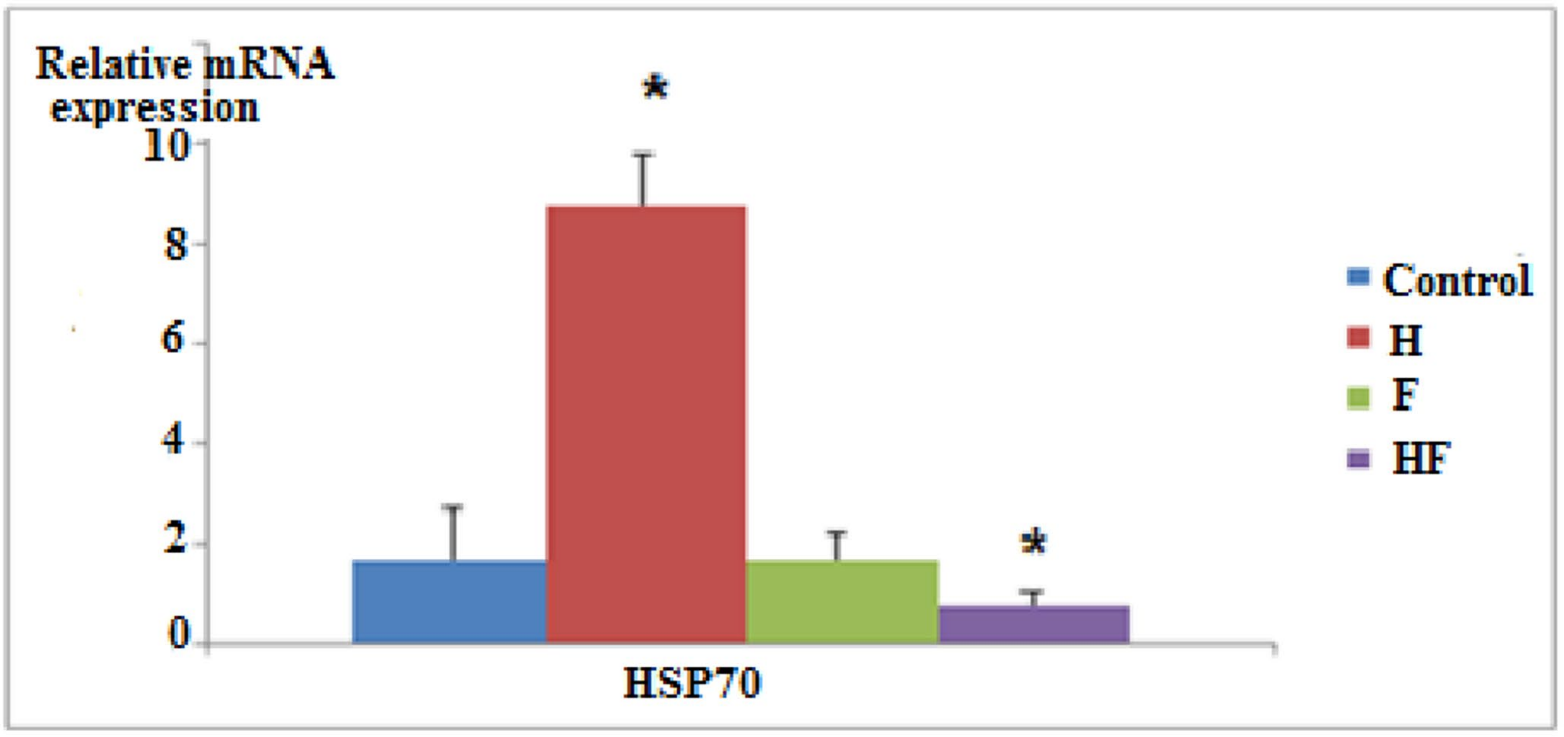
Effect of different administrations on heat shock protein 70 (HSP70) gene expression level. Data are represented as mean ±SD.* P* significant at *P *≤ 0.05. P* significant compared to the normal control group

Expression of (HSP70) gene was significantly up-regulated in the (H) group while significantly down-regulated in HF(H+F) group but it was not recorded significantly differences in (F) group compared to the control group (Fig. 6).

### Molecular docking 

#### Molecular interactions of salts with the amino acids of the target genes

The docking results provide a detailed analysis of the molecular interactions between sodium fluoride, cadmium chloride, and sodium arsenate with (HSP70) and (MT1). 

Table (2)summarizing these interactions highlights the hydrophilic hydrogen bonds and hydrophobic contacts formed with specific amino acid residues, along with their bond lengths, the number of interactions, and the binding affinities measured in kcal/mol.

**Table 2.**
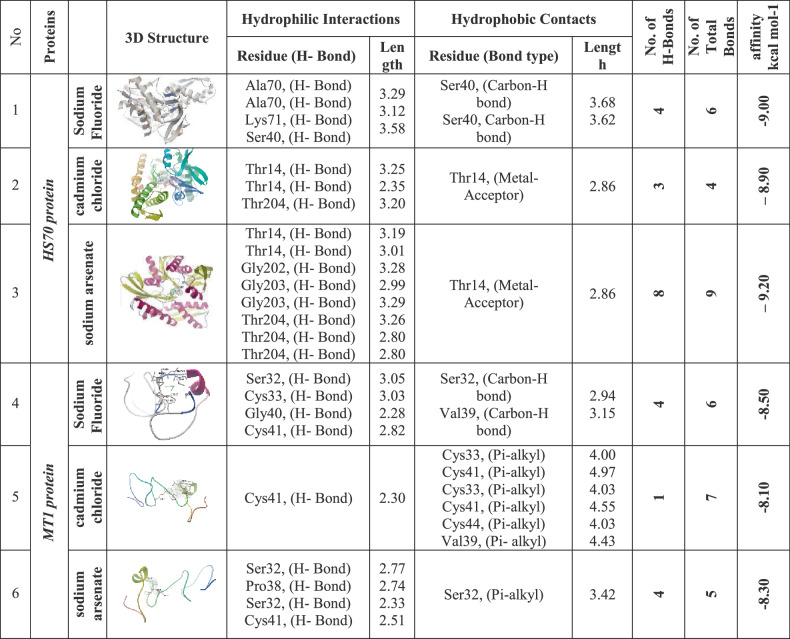
Molecular interactions of salts with amino acids of the list of targets

*Ala *Alanine, *Arg* Arginine, *Asn* Asparagine, *Asp* Aspartic acid, *Cys* Cysteine, *Glu* Glutamic acid, *Gln* Glutamine,*Gly* Glycine, *His* Histidine, *Ile* Isoleucine, *Leu* Leucine, *Lys* Lysine, *Met* Methionine, *Phe* Phenylalanine,*Pro* Proline, *Ser* Serine, *Thr* Threonine, *Trp* Tryptophan, *Tyr* Tyrosine, *Val* Valine

#### Molecular Interactions of Sodium Fluoride with (HSP70) and (MT1)

**Fig. 7:**
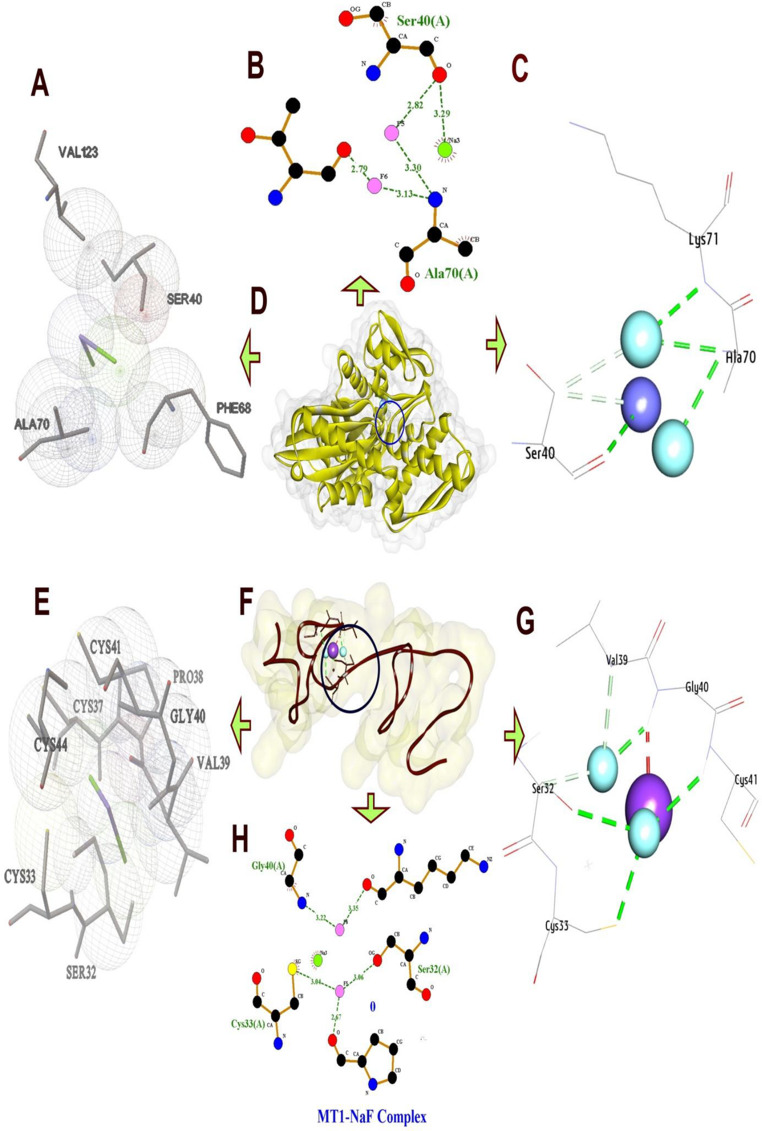
Three-dimensional complex of sodium fluoride conformations at the binding pocket of protein targets: [**A**, **B**, **C**, and **D**] HS70 (PDB:ID 7F50) and [**E**, **F**, **G**, and **H**] MT1 (AF-P80297-F1) the left side showed features interaction between residues and NaF, the right side showed chemical structures of compounds and interacting amino acid residues according to their type

(HSP70), a highly conserved ATP-dependent molecular chaperone, is critical for protein quality control, stress adaptation, and cellular homeostasis. The docking analysis for (HSP70) and (Na F) reveals a robust set of hydrophobic interactions and hydrogen bonds, indicating a stable binding configuration. The 3D structure shows multiple residues involved in hydrogen bonding, including Ala70 (H-Bond, 3.29 Å), Lys71 (H-Bond, 3.12 Å), and Ser74 (H-Bond, 3.58 Å), suggesting a well-defined interaction network with (Na F). These bond lengths are within the typical range for hydrogen bonds (2.5–3.5 Å), indicating strong and specific interactions. Additionally, hydrophobic contacts are observed with Ser40 (Carbon-H bond, 3.68 Å) and Ser40 (Carbon-H bond, 3.62 Å), further stabilizing the complex. The docking score of (−9.00 kcal/mol) reflects a favorable thermodynamic profile. The hydrophobic interactions, though fewer in number, complement the hydrogen bonds by reducing the solvation energy penalty, enhancing overall stability (Fig. 7).

The metallothionein 1 (MT1) gene, which is critical for cellular homeostasis, stress response, and protection against toxicity. The docking analysis for sodium fluoride indicates a stable binding configuration with a mix of hydrogen bonds and hydrophobic interactions, resulting in an affinity of (−8.50 kcal/mol). Hydrogen bonds are formed by Ser32 (H-Bond, 3.05 Å), Cys33 (H-Bond, 3.05 Å), Gly40 (H-Bond, 2.28 Å), and Cys41 (H-Bond, 2.82 Å), with bond lengths within the typical range (2.5–3.5 Å). Hydrophobic contacts include Ser32 (Carbon-H bond, 2.94 Å) and Val39 (Carbon-H bond, 3.15 Å), which enhance stability by reducing solvation energy. The affinity of (−8.50 kcal/mol) reflects a favorable binding energy, driven by the combination of tight hydrogen bonds and hydrophobic contacts. This docking profile implies that sodium fluoride could be involved in a biological function requiring specific polar and hydrophobic stabilization, such as enzyme-substrate recognition, though further studies are needed to elucidate its exact role (Fig. 7).

#### Molecular Interactions of Cadmium Chloride with (HSP70) and (MT1)

**Fig. 8:**
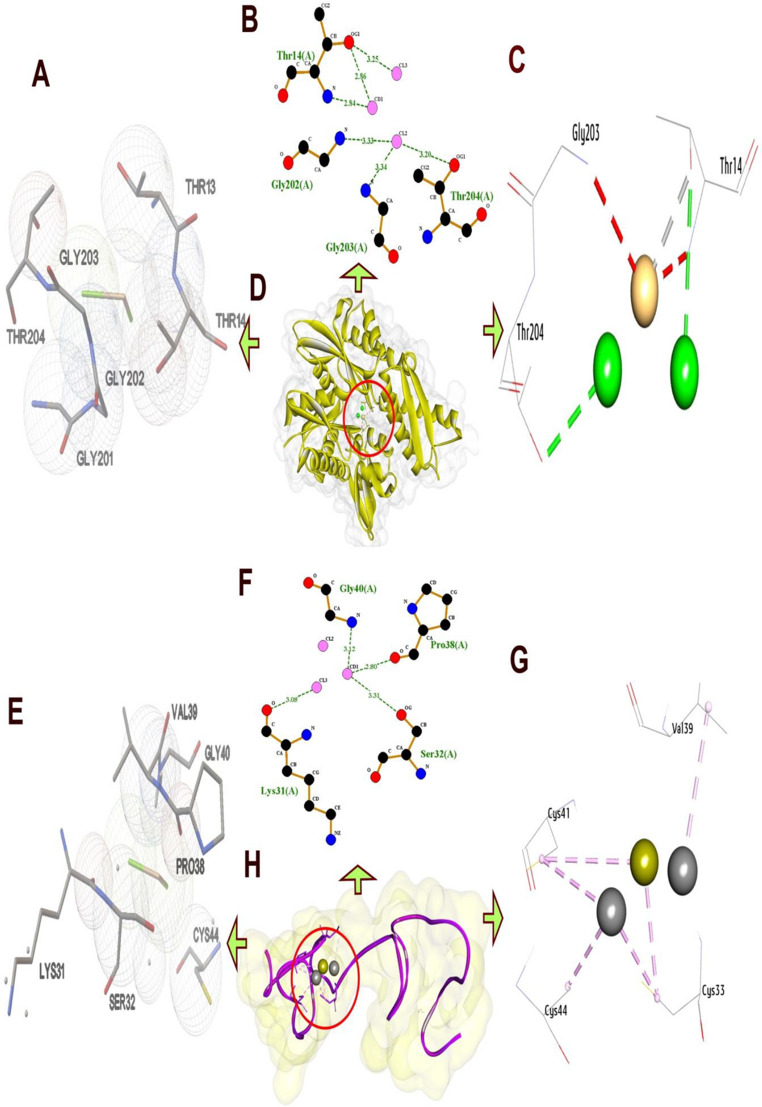
Three-dimensional complex of cadmium chloride conformations at the binding pocket of protein targets: [**A**, **B**, **C**, and **D**] HS70 (PDB:ID 7F50) and [**E**, **F**, **G**, and **H**] MT1 (AF-P80297-F1) the left side showed features interaction between residues and cadmium chloride, the right side showed chemical structures of compounds and interacting amino acid residues according to their type

For (HS70) and cadmium chloride, the docking analysis indicates a diverse interaction profile with a mix of hydrogen bonds and metal-acceptor interactions, contributing to a binding affinity of (−8.90 kcal/mol). The hydrogen bonds involve Thr14 (H-Bond, 3.25 Å), Thr14 (H-Bond, 3.35 Å), and Thr204 (H-Bond, 3.20 Å), with bond lengths consistent with stable hydrogen bonding. Additionally, Thr14 are involved in metal-acceptor interactions (2.86 Å), which are less common but suggest coordination with a metal ion in the active site, potentially enhancing binding specificity (Fig. 8).

Docking analysis for (MT1) and cadmium chloride reveals a binding affinity of (−8.10 kcal/mol), dominated by pi-alkyl interactions with minimal hydrogen bonding. A single hydrogen bond is formed by Cys41 (H-Bond, 2.30 Å), with a bond length indicating a strong interaction. Pi-alkyl interactions are extensive, involving Cys33 (4.00 Å), Cys41 (4.97 Å), Cys43 (4.03 Å), Cys44 (4.55 Å), and Val39 (4.43 Å), which are typical distances for such hydrophobic and aromatic contacts, stabilizing the complex through non-polar forces. The affinity of (−8.10 kcal/mol) suggests a moderate binding strength, largely due to the extensive pi-alkyl network rather than hydrogen bonds. This suggests additional stabilization through aromatic or aliphatic residue contacts, though these distances are longer, typical for such interactions(Fig. 8).

#### Molecular interactions of sodium arsenate with HSP70 and MT1

**Fig. 9:**
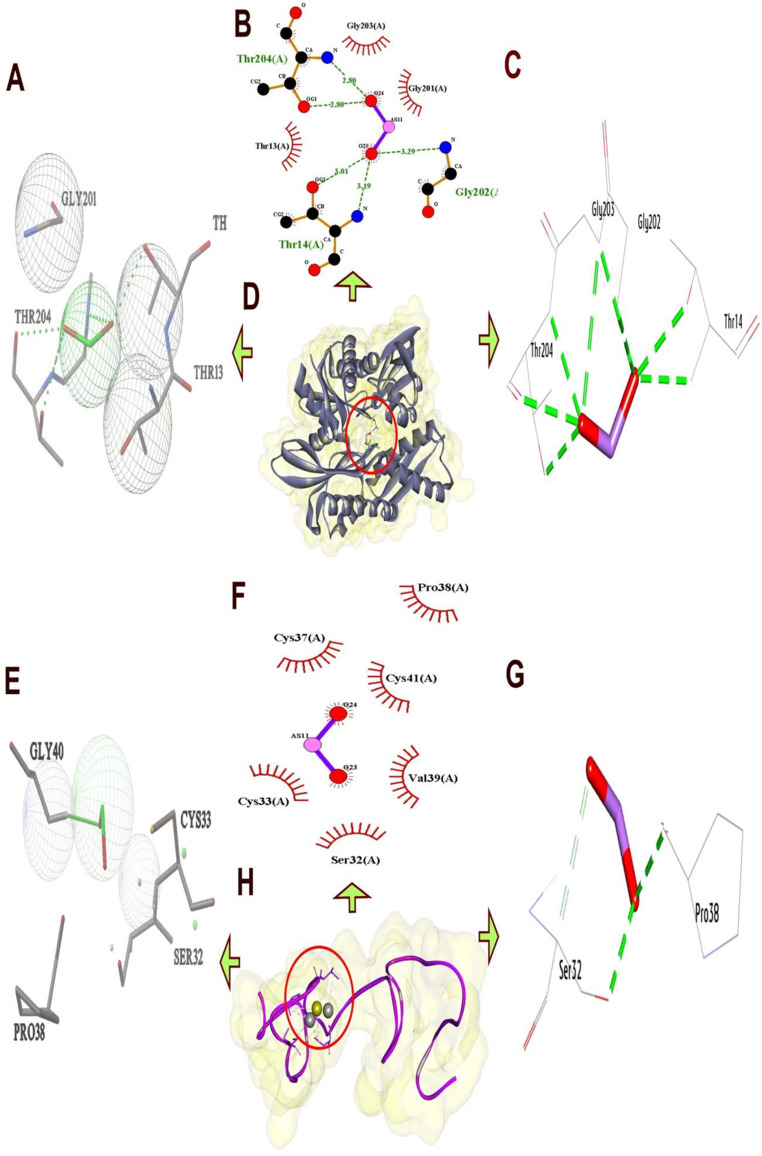
Three-dimensional complex of sodium arsenate conformations at the binding pocket of protein targets: [**A**, **B**, **C**, and **D**] HS70 (PDB:ID 7F50) and [**E**, **F**, **G**, and **H**] MT1 (AF-P80297-F1) the left side showed features interaction between residues and sodium arsenate, the right side showed chemical structures of compounds and interacting amino acid residues according to their type

The docking analysis of (HS70) and sodium arsenate presents a complex interaction pattern with a binding affinity of (−9.20 kcal/mol), indicating strong binding affinity. Hydrogen bonds are formed by Thr14 (H-Bond, 3.19 Å), Gly203 (H-Bond, 3.01 Å), Gly203 (H-Bond, 2.99 Å), Thr204 (H-Bond, 3.26 Å), Thr204 (H-Bond, 3.80 Å), and Ser32 (H-Bond, 3.05 Å), with lengths mostly within the optimal range. Hydrophobic contacts include Thr14 (Metal-Acceptor), 2.86 Å), adding to the stability. The presence of 8 hydrogen bonds and 9 total bonds highlights an extensive interaction network, as visualized in the 3D structure where the ligand is deeply embedded among these residues (Fig. 9). The high affinity of (−9.20 kcal/mol) reflects a thermodynamically favorable binding, likely due to the large number of interactions compensating for the slightly longer Thr204 bond. The combination of numerous hydrogen bonds and hydrophobic contacts indicates that sodium arsenate may play a role in a process requiring tight ligand binding.

The docking analysis for (MT1) and sodium arsenate shows a binding affinity of (−8.30 kcal/mol), supported by a combination of hydrogen bonds and a pi-alkyl interaction. Hydrogen bonds are formed by Ser32 (H-Bond, 2.77 Å), Pro38 (H-Bond, 2.74 Å), Ser41 (H-Bond, 2.83 Å), and Cys41 (H-Bond, 2.51 Å), with bond lengths within the optimal range, particularly the strong 2.51 Å bond of Cys41. A pi-alkyl interaction with Ser32 (3.42 Å) adds additional stability. The total of 4 hydrogen bonds and 5 total bonds suggests compact but effective binding interactions (Fig. 9).

Additionally, the RMSD values from redocking controls (1.33 Å for HSP70 and 1.58 Å for MT1) underscore the accuracy of the docking poses, reinforcing the reliability of the interaction profiles observed.

### Histopathological results 

#### Liver

**Fig. 10:**
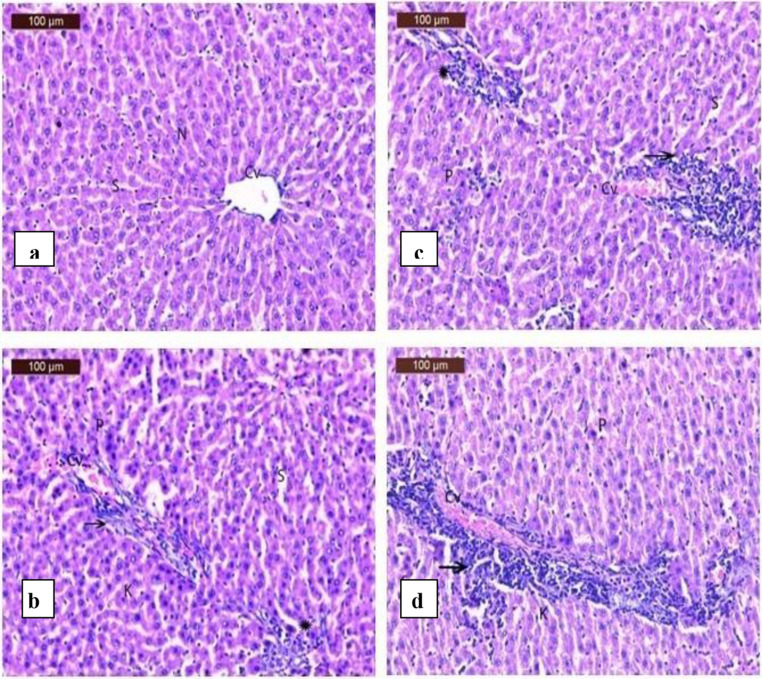
(**a**) A photomicrograph of rat liver in control group showing normal hepatic architecture, central vein (CV), blood sinusoids (S) and nucleus (S). (**b**) A photomicrograph of rat liver in sodium fluoride group showing mild histological structure changes with mild congestion of central vein (Cv), inflammatory cell around central vein (arrow), normal sinusoids (S), few pyknotic nuclei (P), slight Kupffer Cells (K) and slight focal inflammatory cells (star). (**c**) A photomicrograph of rat liver in heavy metals group showing moderate histological structure changes with mild congestion of central vein (Cv) with inflammatory cell around central vein (arrow), normal sinusoids (S), few pyknotic nuclei (P), slight Kupffer cells (K)and slight focal inflammatory cells (star). (**d**) A photomicrograph of rat liver in sodium fluoride and heavy metal group showing moderate histological structure changes with dilated and congestion of central vein (Cv) with sever inflammatory cell around central vein (arrow), normal sinusoids (S), moderate pyknotic nuclei (P) and Kupffer cells (K)

The liver sections of the group administrated sodium fluoride showed mild histological structure changes with mild congestion of central vein, inflammatory cell around central vein, normal sinusoids, few pyknotic nuclei, slight Kupffer cells and slight focal inflammatory cells (Fig.1b).

The liver sections of the group administrated heavy metals showed moderate histological structure changes with mild congestion of central vein, inflammatory cell around central vein, normal sinusoids, few pyknotic nuclei and slight Kupffer cells (Fig.1c).

In the group administrated sodium fluoride and heavy metals, there were a moderate histological structure changes with dilated and congestion of central vein, sever inflammatory cell around central vein, normal sinusoids, moderate pyknotic nuclei and Kupffer cells (Fig.1d).

#### Kidney

**Fig. 11:**
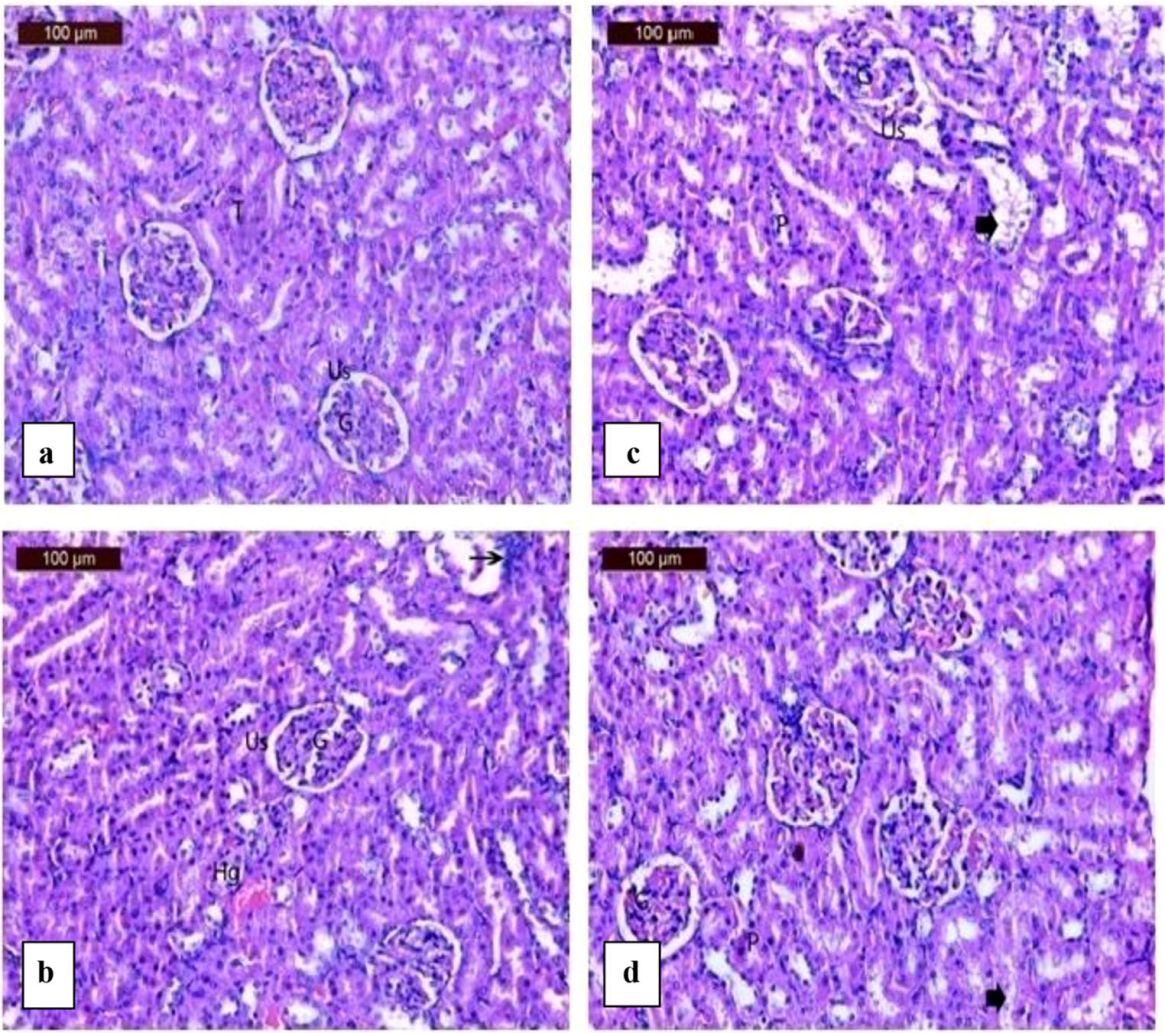
(**a**) A photomicrograph of rat kidney in control group showing normal structure of the glomerulus (G), urinary space (us) and tubules (T). (**b**)A photomicrograph of rat kidney in sodium fluoride group showing mild glomeruli shrunken (G), urinary space (Us), mild inflammatory cells (arrow) interstitial haemorrhage (Hg) and pyknotic nuclei (P). (**c**) A photomicrograph of rat kidney in heavy metal group showing mild glomeruli shrunken (G), urinary space (Us), moderated degeneration of tubules (arrowhead) and pyknotic nuclei (P). (**d**) A photomicrograph of rat kidney in sodium fluoride and heavy metal group showing mild glomeruli shrunken with congestion (G), urinary space (Us), moderated degeneration of tubules (arrowhead) and pyknotic nuclei (P)

The kidney sections of the group administrated sodium fluoride showed mild glomeruli shrunken, urinary space, mild inflammatory cells, interstitial hemorrhage and pyknotic nuclei (Fig. 11b).

The kidney sections of the of heavy metals group showed mild glomeruli shrunken, urinary space, moderated degeneration of tubules and pyknotic nuclei (Fig.11c) In the group administrated sodium fluoride and heavy metals, kidney sections showed mild glomeruli shrunken with congestion, urinary space, moderated degeneration of tubules and pyknotic nuclei (Fig. 11d).

#### Thyroids

**Fig. 12:**
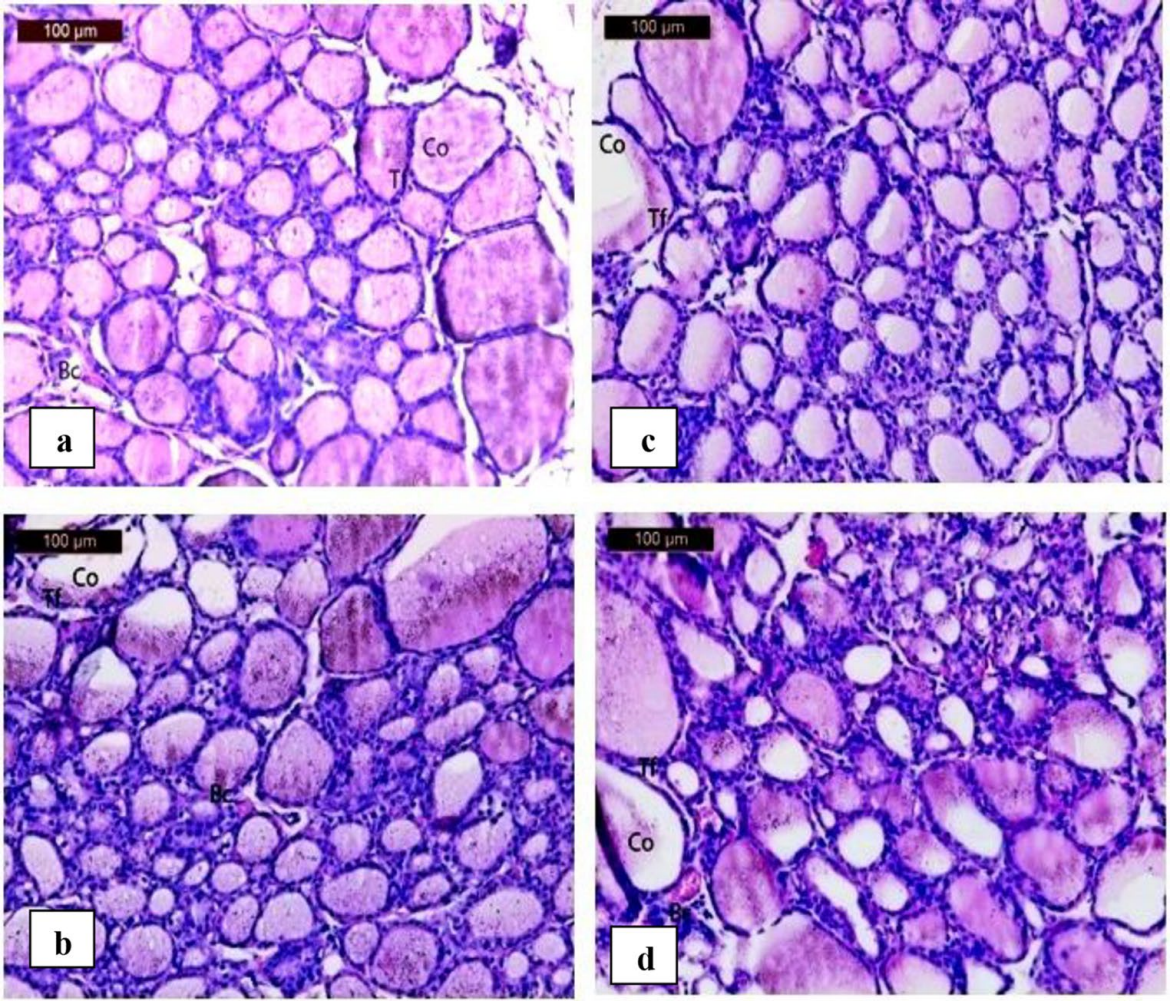
(**a**) A photomicrograph of section of thyroid gland in control group showing normal thyroid architecture with variable follicles lined with cuboidal follicular epithelium (Tf) with rounded nuclei. The follicular lamina is filled with homogenous acidophilic colloid (Co). Normal blood capillaries (Bc) are also found. (**b**) A photomicrograph of rat thyroid gland in sodium fluoride group showing thyroid follicles appeared nearly normal architecture(Tf), the colloid material filled the follicle lumen; and the follicular cells exhibited decrease in colloidal material (Co)Congested blood vessels (Bc) also are seen in thyroid parenchyma. (**c**) A photomicrograph of rat thyroid gland in heavy metal group showing thyroid follicles appeared to have variable forms (Tf), where some follicles appeared shrunken and atrophied, decrease in colloidal material (Co) in some follicles. (**d**) A photomicrograph of rat thyroid gland in sodium fluoride and heavy metal group showing thyroid follicles appeared to have variable forms (Tf), where some follicles appeared shrunken and atrophied, decrease in colloidal material (Co) in some follicles. Congested blood vessels capillaries (BC) also are seen in thyroid parenchyma

The sections of sodium fluoride group showed thyroid follicles appeared to have variable forms, where some follicles appeared shrunken and atrophied, decrease in colloidal material in some follicles. Congested blood vessels also are seen in thyroid parenchyma (Fig.1b).

The sections of heavy metals group showed thyroid follicles appeared nearly normal architecture, the colloid material filled the follicle lumen; and the follicular cells exhibited decrease in colloidal material. Congested blood vessels also are seen in thyroid parenchyma (Fig.12c).

The sections of sodium fluoride and heavy metals group showed different sizes of thyroid follicles, shrunked and atropic follicles were observed. A decrease in colloidal material was noticed in some follicles besides the presence of congested blood vessels in thyroid parenchyma (Fig.12d).

#### Bone marrow 

**Fig. 13:**
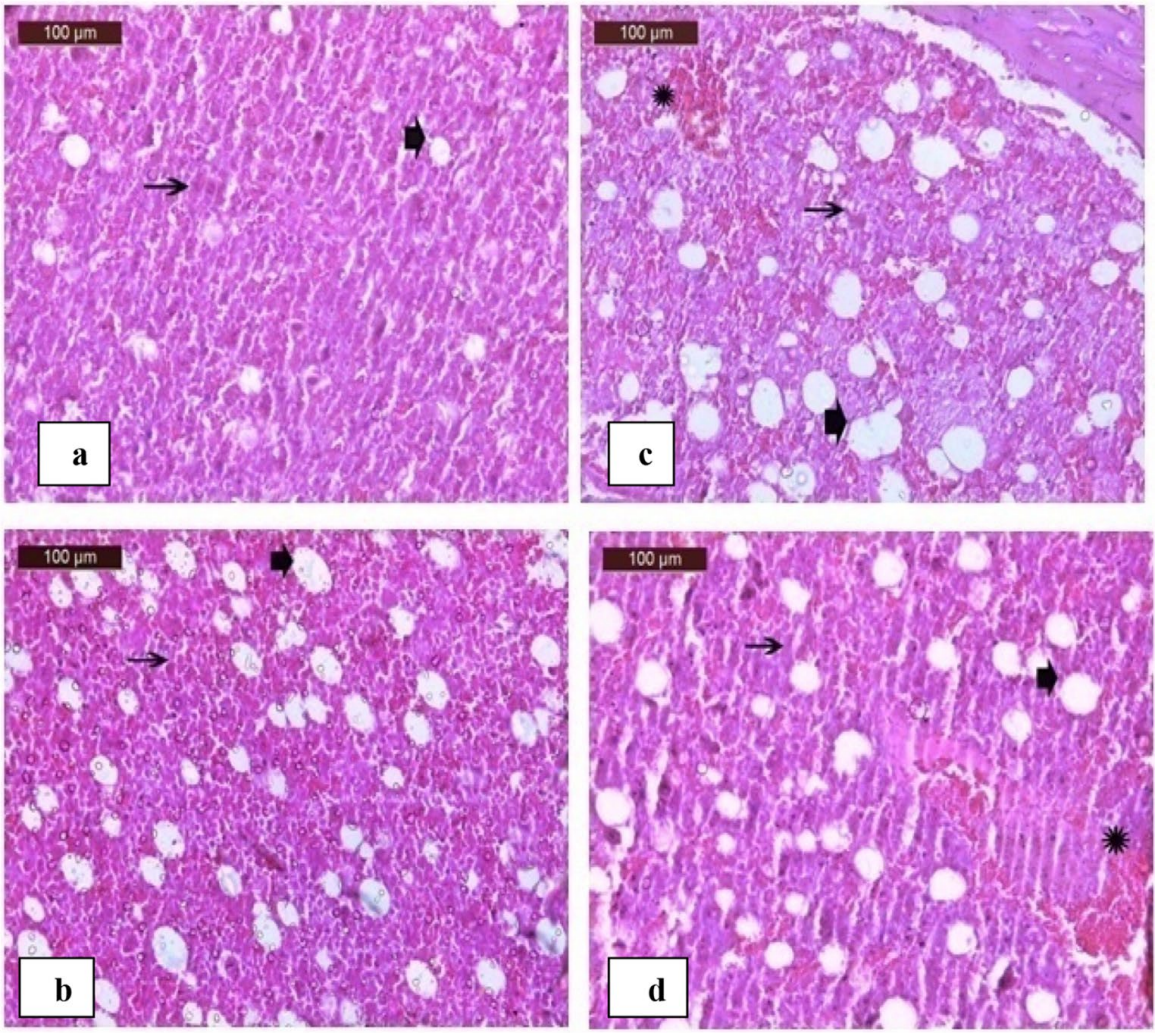
(**a**) A photomicrograph of rat bone marrow in control group showing normal bone marrow, cellular tissue (black arrows) and adipose tissue (arrowhead).(**b**) A photomicrograph of rat bone marrow in sodium fluoride group showing mild increase in adipose tissue in bone marrow (arrowhead) and mild decreased cellular tissue (arrows). (**c**) A photomicrograph of rat bone marrow in heavy metal group showing moderate increase in adipose tissue in bone marrow (arrowhead), decreased cellular tissue (arrows) and mild hemorrhage (Star). (**d**) A photomicrograph of rat bone marrow in sodium fluoride and heavy metal group showing moderate increase in adipose tissue in bone marrow (arrowhead), decreased cellular tissue (arrows) and moderate hemorrhage (Star)

The bone marrow sections of the group administrated sodium fluoride showed mild increase in adipose tissue in bone marrow and mild decreased cellular tissue (Fig.13b) The bone marrow sections of the group administrated heavy metals showed moderate increase in adipose tissue in bone marrow, decreased cellular tissue and mild hemorrhage (Fig.13c).

In the group administrated with sodium fluoride and heavy metals, moderate increase in adipose tissue in bone marrow, decreased cellular tissue and moderate hemorrhage w In the group administrated with sodium fluoride and heavy metals, moderate increase in adipose tissue in bone marrow, decreased cellular tissue and moderate hemorrhage were found (Fig.13d).

Semi-quantitative analysis of histological findings in liver, kidneys, thyroid glands and bone marrow in different rat groups were reported in the present study (Supplementary tables (S6-S9)).

## Discussion

Cadmium, arsenic, fluoride, and lead are among the most well-known environmental pollutants that can cause numerous complications in various body organs in humans and animals [40].

The effect of the heavy metals or non metals on the organs functions depends on many factors including the dose, the route of administration and the interactions between the administrated compounds [54–56].

In the present study, the body weight in the different administrated groups (F, H and (H + F) groups) was affected and increased significantly compared to the control group after one and two months (Figs. 1(a) & (2)). The weight gain is influenced by how well the nutrients were absorbed and used by the body [57]. Exposure to cadmium, arsenic combination and/or fluoride lead to the accumulation of these substances in the tissues of rats thus disrupted several vital processes that affect the metabolism such as changing the microbial environment in the intestine, altering intestinal mucosal permeability and changing the intestinal metallothionine expression besides localization and distribution of glycan residues in the intestinal cells [58] which in turn affected gradually the weight gain.

Hematological parameters serve as essential indicators and tools for identifying the harmful impacts of toxic substances on an animal’s blood components. These toxic compounds can interfere with blood elements and modify tissue products, resulting in changes in the activity of blood-related parameters [59, 60].

In the current study, histological toxicity signs were observed in the bone marrow sections. These signs were interpreted to significant changes in blood cells count. Significant changes in hematological parameters were observed in the three groups: (F), (H), and (H + F) groups (Table 1). The current study detected mild but significant alterations in blood parameters across the three experimental groups, with one exception which was the platelet (PLT) count. With respect to the control group, (PLTs) count was the most affected parameter, showing a significant increase following exposure to cadmium, arsenic, and/or fluoride (Table 1).

An elevated platelet count, known as thrombocytosis, poses a serious risk to both human and animal health by increasing the likelihood of blood clot formation within vessels. Earlier research has identified platelets as a primary target for heavy metals, such as cadmium and arsenic, often leading to a reduction in platelet count (thrombocytopenia) and in some cases platelet death [61]. These findings did not agree with the outcomes of the current study. The rise in platelet numbers observed in this study may be due to the presence of older platelets remaining in circulation to compensate the existed decline in platelet production from the affected bone marrow, as illustrated in Fig. 13(a, b, c, and d). (Supplementary table (S9)).

The deteriorated condition of the endothelial vasculature and cardiac function, resulting from exposure to cadmium, arsenic, and/or fluoride (to be discussed later), increased the need for more circulating platelets to repair damaged tissues, promoting platelet aggregation. The average lifespan of platelets is about 7 to 10 days, and certain conditions can change their lifespan and rate of turnover [62]. Since platelets are primarily produced in the bone marrow, any disorders in the bone marrow can impact platelet levels [63, 64].

Another key sign of toxicity seen in this study was the unusual levels of some enzymes and hormones in the blood (Figs. 3(a), (b) & (4)), which showed problems with the liver, heart, and thyroid gland [65, 66].

The current study showed that cadmium, arsenic, and/or fluoride caused liver damage, as seen in Fig. 10 (a, b, c, and d) (Supplementary tables (S4) & (S6)).

This damage was reflected in the significant changed levels of ALT, AST, ALP in rats’ serum of different groups. These results matches with those reported in previous studies [67–71].

To study the liver damage in depth, the present study evaluated the expression levels of two important genes closely related to stress and chelating of heavy metals in liver tissue represented in heat shock protein 70 and metallothionine 1 which will be discussed in details further. No signs of kidney problems were found in the current study. During the two-month experiment, the levels of creatinine and urea in the blood stayed normal in the three groups (F), (H), and (H + F) compared to the control group (Fig. 3(b)). Histological examination of the kidney tissue showed mild degeneration of tubule walls, shrunken glomeruli, and disrupted nuclei as demonstrated in Fig. 11 (a, b, c and d). (Supplementary table (S7)).

The present study also predicted a deterioration in cardiac functions, particularly in the (F) group and (H) group, in the short run (Fig. 3(a)). (Supplementary table (S4)).

The present study reported a highly significant increase in serum CPK and LDH, as well as a significant increase in serum AST levels, suggesting a prompt, noxious impact of sodium fluoride on cardiac functions in the (F) group, potentially leading to a future heart failure [72–74].

The results also showed a significant decrease in serum triglycerides (TG) in the (H) group, which can be considered as an indicator for a case of cardiomyopathy, as the cardiomyocytes express high levels of lipoprotein lipase on their surface, leading to increased lipid (TG) consumption [75, 76]. The present study suggested that the combination of cadmium and arsenic salts in the (H) group may have an impact on the relaxation of heart muscle, leading to this pathological condition. These results agreed with those reported by Saroj et al. (2017) and Ohiagu et al. (2022) [77, 78] who found that the administration of heavy metals can cause muscular and neurological abnormalities. The harmful effects of cadmium and arsenic salts on cardiac tissue, even at low doses, have been documented in several studies [9, 79–84].

The administration of fluoride and heavy metals can also disrupt the thyroid tissue, leading to the substitution of beneficial elements, such as iodine, zinc, and selenium, and causing thyroid damage [85]. Disruption of serum thyroid hormone levels can significantly impact heart status [86–90], as observed in the (F) group, which exhibited the most deteriorated predicted heart condition associated with a hypothyroidism in the present work (Fig. 3). (Supplementary table (S5)).

In the (H) and (H + F) groups, serum (FT3) showed significant alterations, while (FT4) levels were not significantly affected, indicating thyroid hormone disruption and expecting later gland dysfunction as illustrated in Fig. 12 (a, b, c and d).

The up regulation of certain genes, such as metallothionein (MT) and heat shock protein (HSP), can also serve as indicators for toxicity [91, 92]. (MT) is considered a potent biomarker of environmental contamination, as its increased expression represents a defensive mechanism in tissues to chelate and control heavy metal levels [5, 93–97]. (HSP) gene expression is up regulated in response to various stressors, including heat, chemical toxins, heavy metals, oxidants, and viruses, and plays a role in protein stability, resistance to apoptosis, and stress control [98–100].

In the present study, the examination of toxicity levels in liver tissue was performed by determining the molecular gene expression levels of (MT1) and (HSP70). The results showed a significant up regulation of (MT1) and (HSP70) in the (H) group. Interestingly, there was a significant up regulation of (MT1) in the (F) group, while (HSP70) was not affected. In contrast, the (H + F) group exhibited a significant down regulation of both genes compared to the control group (Figs. (5) & (6)).

The present study is the first to explore the relationship between (MT1) gene expression in the liver and fluoride administration. The up regulation of (MT1) in the (F) group may be attributed to the stress exerted by sodium fluoride on hepatocytes, causing a decrease in antioxidant levels [101], and inducing endoplasmic reticulum stress and apoptosis [102].

The significant down regulation of both (MT1) and (HSP70) in the (H + F) group may indicate the lethal effect of the administered heavy metals and fluoride mixture on the defense mechanisms in rat liver cells. This can be interpreted in light of previous studies that have linked the down regulation of (MT1) to the stimulation of NF-κB activity, leading to hepatic cancer [103] and the decreased levels of MT1E as a possible biomarker for the advancement of prostate cancer [104–106].

The down regulation of (HSP70) in the (H + F) group is considered as another indicator for showing the low defense mechanisms of hepatocytes against toxicity stress in this group.

(HSP70) down regulation may affect cell integrity [107] and can initiate liver fibrosis while increased levels in the early stages can improve the condition and prevent the development of liver cancer [108–110].

The present study explained for the first time the molecular interactions between the administrated toxic salts and (HSP70) and (MT1) genes’ proteins in order to shed the light on their binding affinities to each other by using the docking analysis tool.

Molecular docking is frequently utilized to forecast the interactions between small-molecule pharmaceuticals and their target proteins, offering estimates of binding affinity and effectiveness. This method is crucial for logical drug development [111–115].

Docking analysis of the genes unveiled that sodium fluoride formed stable complexes with both proteins through a combination of hydrogen bonds and hydrophobic interactions, exhibiting binding affinities of (−9.00 kcal/mol) for (HSP70) and (−8.50 kcal/mol) for (MT1). Cadmium chloride showed distinct binding profiles, engaging (HSP70) at (−8.90 kcal/mol) and (MT1) at (−8.10 kcal/mol). Finally, the binding profiles of sodium arsenate for (HSP70) was (−9.20 kcal/mol) and for (MT1) was (−8.30 kcal/mol). Notably, the combined effects of these compounds on each gene, suggest synergistic inhibition for the expression of the gene (in HSP70 and MT1).

So the docking analyses in the present study revealed that sodium fluoride, cadmium chloride and sodium arsenate exhibit distinct yet complementary binding mechanisms with (HSP70) and (MT1).When all three salts (sodium fluoride, cadmium chloride, and sodium arsenate) are mixed, their distinct interaction profiles could collectively overwhelm (HSP70)’s binding sites. Sodium fluoride and sodium arsenate would saturate polar residues, while cadmium chloride introduces metal-mediated distortions. This multi-targeted approach may potently inhibit (HSP70) by preventing conformational changes essential for its chaperone function, ultimately disrupting cellular stress responses and promoting protein misfolding, a strategy for toxicology studies.

### Limitations of the Study

The present study has certain limitations that should be acknowledged. The experiment was conducted on a relatively small number of female Wistar rats over a two-month exposure period, which may not fully represent the long-term or sex-dependent effects of cadmium, arsenic, and fluoride toxicity. Although the research combined biochemical, histopathological, and molecular approaches, it did not explore other potential biomarkers or signaling pathways that could further explain the observed toxic interactions. Additionally, the doses used, while moderate, may not directly mimic chronic low-level environmental exposure in humans. Finally, the study focused on controlled laboratory conditions, which differ from real environmental settings where multiple pollutants and lifestyle factors interact simultaneously. Future studies are needed to validate these findings under longer exposure durations, with larger sample sizes, and through the inclusion of natural chelating agents or protective dietary interventions.

## Conclusion

Heavy metals and fluoride mixture administration may have a more detrimental impact on health compared to the effects of fluoride or the combination of heavy metals alone. This phenomenon can be linked to its ability to weaken the liver cells’ defense mechanisms by causing protein misfolding and disrupting essential biological processes, which in turn increases the liver diseases and accelerates mortality (Fig. 1(b).

## Supplementary Information

Below is the link to the electronic supplementary material.

Supplementary File 1 (DOCX 35.3 KB)

## Data Availability

All the data obtained in the manuscript is available upon request.

## Abbreviations

ALP: Alkaline phosphatase enzyme
ALT: Alanine aminotransferase enzyme
AST: Aspartate aminotransferase enzyme
As: Arsenic element
Cd: Cadmium element
CPK: Creatine phosphokinase enzyme
DNA: Deoxyriboneucleic acid
EDTA: Ethylene Diamine Tetra Acetic acid
ELISA: Enzyme Linked Immuno Sorbent Assay
F: Fluorine element
FT4: Free Thyroxine hormone
FT3: Free tri-iodothyronine hormone
HSP70: Heat shock protein 70
LDH: Lactate dehydrogenase enzyme
MT1: Metallothionine 1
PLT(s): Platelet
r.p.m: Round per minute
TG: Triglycerides
