# Correction: Bio and Geno-toxic Activities of Cadmium- Arsenic Salts Combination And/or Fluoride in Female Rats Confirmed by Molecular Docking

**DOI:** 10.1007/s12011-026-05068-8

**Published:** 2026-04-07

**Authors:** Doaa S. Foda, Noha E. Ibrahim

**Affiliations:** 1 Therapeutic Chemistry Department, Pharmaceutical and Drug Industries Research Institute, National Research Centre (NRC), 33 El-Buhouth St, Dokki, Giza, P.O. 12622 Egypt; 2 Microbial Biotechnology Department, Biotechnology Research Institute, National Research Centre (NRC), 33 El-Buhouth St, Dokki, Giza, P.O.12622 Egypt

**Correction:** **Biological Trace Element Research**

10.1007/s12011-025-04972-9

The published version of this article unfortunately contained mistakes.

Figure 1 caption should read Fig. 1 (a) Effect of heavy metal combination and /or fluoride on body weights and mortality rate in female rats. Note: Data represented as mean ± S.D. P significant at P ≤ 0.05, P* significant compared to the corresponding initial weight, P# significant compared to the corresponding final normal control groups, P& significant compared to the corresponding final weight after 1 month. (b) Effect of heavy metal combination and /or fluoride on mortality rate in rat groups. Note: Showing the survival and mortality rates (%) across rat groups during two months of different administrations.

Figure 2 caption should be Percent change values in rats' weights after 1 and 2 months representing the percent change in body weights of rats in the different groups compared to their initial corresponding body weights.

Figure 3 caption should be Effect of two months administration of heavy metals combination and/or fluoride on some serum biochemical parameters in female rats. (a): Effect of different administrations on some serum enzymes. Footnote: Data was represented as mean ±S.D. P significant at P ≤ 0.05. P* significant compared to the normal control group. P# significant compared to (H+F) group. (b): Impact of the different administrations on lipid profile and kidney functions. Data is represented as mean ±S.D. P significant at P ≤ 0.05. P* significant compared to the normal control group. P# significant compared to (H+F) group.

Figure 4 caption should be Effect of two months administration of heavy metals combination and/or fluoride on serum FT4 and FT3 in female rats. Data is represented as mean ±S.D. P significant at P ≤ 0.05. P* significant compared to the normal control group. P# significant compared to (H+F) group.

Figure 5 caption should be Effect of different administrations on metallothionine 1 (MT1) gene expression level. Data are represented as mean±SD. P significant at P ≤ 0.05. P* significant compared to the normal control group.

The original article has been corrected.

