# Supplementary material for: Bio and Geno-toxic Activities of Cadmium- Arsenic Salts Combination And/or Fluoride in Female Rats Confirmed by Molecular Docking
Source: Biol Trace Elem Res. 2026 Feb 24;204(6):4391–414. doi: 10.1007/s12011-025-04972-9 (PMC13157369; doi:10.1007/s12011-025-04972-9)
Supplement: Supplementary file 1 — (DOCX 35.3 KB) [file 12011_2025_4972_MOESM1_ESM.docx]

**Supplementary file**

**(Ref: Submission ID 30a9fa42-fc94-4541-87f0-191031affc28 )**

**Bio and geno-toxic activities of cadmium-** **arsenic salts combination and/ or fluoride in female rats confirmed by molecular docking.**

Doaa S Foda ^1*^ and Noha E Ibrahim^2^.

^1^Therapeutic Chemistry Department, Pharmaceutical and Drug Industries Research Inistitute, National Research Centre (NRC), 33 El-Buhouth St., Dokki, Giza, P.O. 12622, Egypt.

^2^Microbial Biotechnology Department, Biotechnology Research Institute, National Research Centre (NRC), 33 El-Buhouth St., Dokki, Giza, P.O. 12622, Dokki, Giza, Egypt.

**Corresponding author:** Doaa S Foda.

**E- mail:** d.foda2018@gmail.com

**Materials and methods**

**Table (S1): The sequences of the primers of MT1 and HSP70 genes.**

| **References** | **Product length** | **Primer sequence** | **Primer name** | **No.** |
| --- | --- | --- | --- | --- |
| [49] | 310 | F: ACTGCCTTCTTGTCGCTTA  R: TGGAGGTGTACGGCAAGACT | *Rattus norvegicus* metallothionein 1 (MT1) | 1 |
| [50] | 491 | F: TGCTGACCAAGATGAAG  R: AGAGTCGATCTCCAGGC | *Rattus norvegicus* heat shock protein family A (Hsp70) member 1A (HSPa1a) | 2 |
| [51] | 138 | F: CATTGCTGACAGGATGCAGAAGG  R: TGCTGGAAGGTGGACAGTGAGG | *Rattus norvegicus* actin, beta (Actb) | 3 |

**Table (S2): List of targets, PDB IDs, resolution, and active site coordinates of docking analysis.**

| **NO** | **Protein Targets** | **PDB ID** | **Resolution** | **Active site coordinates:** | | | **Native ligands** | **RMSD**  **Values** | **Reference** |
| --- | --- | --- | --- | --- | --- | --- | --- | --- | --- |
|  |  |  |  | **Center-X**  **Size-X** | **Center-Y**  **Size-Y** | **Center-Z**  **Size-Z** |  |  |  |
| 1 | HSP70 | **7F50** | 1.70 Å | -6.27  (60 Å) | 13.35  (60 Å) | -18.55  (60 Å) | ANP | 1.33 Å | [52] |
| **2** | MT1 | **AF-P80297-F1** | 2.02 Å | -5.50  (60 Å) | 3.64  (60 Å) | 7.50  (60 Å) | Zn²⁺-bound | 1.58 Å | - |

**Results**

**Table (S3):**

**Effect of different administrations on body weights and mortality rate in female rats.**

| **Parameters**  **Groups (10 rats each)** | **Initial weight**  **(grams)** | **Final weight after 1 month** | **Final weight after 2 months** | **Mortality rate** |
| --- | --- | --- | --- | --- |
| **Fluoride (F) only** | **105.04±7.59** | **162.92±18.21*** | **213.50±28.45*^,#^**^, &^ | **0/10** |
| **Heavy metals combination**  **(H)** | **95±4.34** | **173.83±13.7*^,#^** | **191.67±13.66***^,&^ | **0/10** |
| **Heavy metals and fluoride mixture (H+F)** | **97.75±6.95** | **192.17±15.98*^,#^** | **214.17±16.40*^,#^**^,&^ | **4 /10**  **(40%)** |
| **Normal**  **Control** | **97.38±5** | **156.73±4.26*** | **200±6.13***^,&^ | **0/10** |

Data represented as mean ± S.D. *P* significant at *P* ≤ 0.05, *P** significant compared to the corresponding initial weight, *P*^#^ significant compared to the corresponding final normal control groups, *P*^&^ significant compared to the corresponding final weight after 1 month.

**Table (S4): Effect of two months administration of heavy metals combination and/fluoride on some serum biochemical parameters in female rats.**

| **Groups**  **Parameters** | **Fluoride** | **Heavy metals** | **Heavy metals**  **and Fluoride** | **Control** |
| --- | --- | --- | --- | --- |
| **ALT (U/L)** | **33.5±0.5*,#** | **51±2*,#** | **42±2*** | **36.5±0.5** |
| **AST (U/L)** | **131±9*,#** | **115±9** | **110±12** | **101±7.5** |
| **ALP (U/L)** | **56±1*,#** | **101±2*,#** | **75±7*** | **86±2.5** |
| **LDH (U/L)** | **1760±520*, #** | **590±230** | **530±230** | **910±130** |
| **Cholesterol**  **(mg/dl)** | **71.50±4.5** | **65±3** | **69±2.5** | **67±2** |
| **Triglycerides**  **(mg/dl)** | **122.5±21.5** | **41±2*,#** | **127.5±16.5** | **116.5±21.5** |
| **Urea (mg/dl)** | **36.5±3.5** | **43±9** | **45.5±3.5** | **39.5±0.5** |
| **Creatinine**  **(mg/dl)** | **0.55±0.05** | **0.5±0.01** | **0.5±0.1** | **0.5±0.1** |
| **Creatine phospho- kinase (CPK)**  **(U/L)** | **442±44*#** | **169.3±9.5*#** | **282.3±73.5** | **278.3±12.50** |

Data is represented as mean ±S.D. *P* significant at *P* ≤ 0.05. *P** significant compared to the normal control group. *P*^#^ significant compared to (H+F) group.

**Table (S5): Effect of two months administration of heavy metals combination and/or fluoride on serum FT4and FT3 in female rats.**

| **Groups**  **Parameters** | **Fluoride** | **Heavy metals** | **Heavy metals and Fluoride** | **Control** |
| --- | --- | --- | --- | --- |
| **FT4**  **(ng/dl)** | **1.27±0.02*#** | **1.86±0.08** | **2.04±0.18** | **1.77±0.33** |
| **FT3**  **(pg/ml)** | **2.23±0.02*#** | **2.61±0.13*#** | **3.77±0.095*** | **3.28±0.14** |

Data is represented as mean ±S.D. *P* significant at *P* ≤ 0.05. *P** significant compared to the normal control group. *P^#^* significant compared to fluoride and heavy metal mixture group.

**Semi-quantitative analysis of histological findings:**

Histological changes scored on a 4-point scale: (-) none, (+) mild, (++) moderate, and (+++) severe damage. A minimum of 10 fields for each section of slide were examined and assigned for severity of changes using scores.

**Table (S6): Semi-quantitative recording of architectural damage on histopathological analysis of livers in different rat groups.**

| **Parameters** | **Histological changes** | **Pyknotic nuclei** | **Dilated central vein congested** | **Kupfer cells** | **Inflammatory cells** | **Dilated sinusoids** |
| --- | --- | --- | --- | --- | --- | --- |
| **Control** | **-** | **-** | **-** | **-** | **-** | **-** |
| **sodium fluoride** | **+** | **+** | **+** | **+** | **+** | **-** |
| **heavy metal** | **++** | **+** | **+** | **+** | **++** | **-** |
| **sodium fluoride + heavy metal** | **++** | **++** | **++** | **+** | **+++** | **-** |

Note: ^__,^ no changes; + slight changes; ++ moderate changes; +++ severe changes.

**Table (S7): Semi-quantitative recording of architectural damage on histopathological analysis of kidneys in different rat groups.**

| **Parameters** | **Shrunken of glomeruli** | **Dilated urinary space** | **Tubular epithelial cell degeneration** | **Interstitial**  **hemorrhage** | **Pyknotic nuclei** |
| --- | --- | --- | --- | --- | --- |
| **Control** | **-** | **-** | **-** | **-** | **-** |
| **sodium fluoride** | **+** | **-** | **-** | **+** | **+** |
| **heavy metal** | **+** | **+** | **++** | **-** | **+** |
| **sodium fluoride + heavy metal** | **+** | **+** | **++** | **-** | **+** |

Note: ^__,^ no changes; + slight changes; ++ moderate changes; +++ severe changes.

**Table (S8): Semi-quantitative recording of architectural damage on histopathological analysis of thyroid glands in different rat groups.**

| **Parameters** | **Follicles shrunken and atrophied** | **Decrease in colloidal material** | **Dilated central vein congested** |
| --- | --- | --- | --- |
| **Control** | **-** | **-** | **-** |
| **sodium fluoride** | **-** | **+** | **+** |
| **heavy metal** | **+** | **++** | **-** |
| **sodium fluoride + heavy metal** | **+** | **++** | **++** |

Note: ^__,^ no changes; + slight changes; ++ moderate changes; +++ severe changes.

**Table (S9): Semi-quantitative recording of architectural damage on histopathological analysis of bone marrow in rat groups.**

| **Parameters** | **Adipose tissue in bone marrow** | **Decreased cellular tissue** | **Hemorrhage** |
| --- | --- | --- | --- |
| **Control** | **-** | **-** | **-** |
| **sodium fluoride** | **+** | **+** | **-** |
| **heavy metal** | **++** | **++** | **+** |
| **sodium fluoride + heavy metal** | **++** | **++** | **++** |

Note: ^__,^ no changes; + slight changes; ++ moderate changes; +++ severe changes.
